# Supplementary material for: Plant growth regulators interact with elevated temperature to alter heat stress signaling via the Unfolded Protein Response in maize
Source: Sci Rep. 2019 Jul 17;9:10392. doi: 10.1038/s41598-019-46839-9 (PMC6637120; doi:10.1038/s41598-019-46839-9)
Supplement: Supplementary file 1 — Supplemental File Information [file 41598_2019_46839_MOESM1_ESM.docx]

**Supplemental File Information for:**

**Plant growth regulators interact with elevated temperature to alter heat stress signaling via the Unfolded Protein Response in maize.**

Elena M. Neill, Michael C. R. Byrd, Thomas Billman, Federica Brandizzi, and Ann

E. Stapleton

All supplemental files are available at DOI 10.6084/m9.figshare.7609931, [https:](https://figshare.com/s/175af4c449c8ccfe9a64)

[//figshare.com/s/175af4c449c8ccfe9a64](https://figshare.com/s/175af4c449c8ccfe9a64).

The metadata for each file is in the figshare repository and is also included below.

**Metadata for Neill et al Supplemental Files**

Each supplemental file name is listed with text explanations of the file content below the name. If data is provided in the file, the columns and row IDs are listed and defined.

Supplemental Methods File1 July14_ire_bzip qPCR design sequences.docx

Annotated, color-coded maize bZIP60 sequence, to illustrate primer and probe design constraints. We avoided highly polymorphic SNPs by checking for matches to a newly sequenced inbred (PH207).

Supplemental Methods File2 7 Mar 2018 realtime plate map.doc

This is a diagram of which sample was placed in which well of the realtime PCR instrument. The test2 reactions are not relevant to this work. The “+IRE” primer listed in this file is named “all_products” in our Table 1 and throughout the manuscript text and additional results files.

Supplemental Results File1 RNA test 7 Mar 2018 Quantification Amplification Results.xlsx

This is the raw fluorescence counts from the realtime PCR instrument for each cycle. The column number-letter ID maps to the sample number in the Supplemental Methods File2 7 Mar 2018 realtime plate map.doc file.

Supplemental Results File2 vNov18Neill thesis plant measurements.xlsx

The column header for each column in this file is explained below:

**Temperature** = the temperature applied during the differential portion of the experiment, where “hot” refers to the high-temperature chamber and “normal” refers to the control chamber with an unchanged temperature regime.

**Treatment** = plant growth regulator application code, with the untreated control named as “no hormone”. All plant growth regulators were applied at 50 ppm, with 10 milliliters applied to each plant pot.

**Plant** = the plant within each pot, there are five (n=5) for each treatment-temperature combination.

**SampleID_RNA** = first leaf sample unique ID, with the treatment abbreviation, underscore, replicate (as a letter of the alphabet), underscore, then a unique sample number.

**Sample2_ID_RNA** = second (taken adjacent to first) leaf sample unique ID, with the treatment abbreviation, underscore, replicate (as a letter of the alphabet), underscore, then a unique sample number.

**Aboveground Stem Height (cm)** = height of plant measured from soil surface to insertion of the top-most leaf, in centimeters. This is abbreviated as ‘plant height’ in the manuscript.

**Total Biomass (g)** = sum of Stem Leaf Dry Weight (g) and Root Mass (dry weight in grams), in units of grams.

**Stem Leaf Dry Weight (g)** = Weight of dried above-ground tissue (after the RNA sample leaf slices of equal size were removed from each plant), in units of grams.

**Root Mass (dry weight in grams)** = Weight of root tissue after rinsing to remove loose soil particles and full drying.

Supplemental Results File3 Neill2018calibrations.xlsx

The column header for each column in this file is explained below. Comparison of the Cp values from test_1 and all_products illustrates the difference in reaction efficiency of these two primer sets.

*replicate* = letter code for the three replicates of each input gblocks synthetic template amount

*amount_gblock+ire* = amount (in picograms) of the input gBlock template (listed in Table 1 with the label “gblock plus18IRE”). This gBlock sequence is expect to be a template for both the test1 and the all_products primer sets.

*test1_Cp1* = first derivative of the five-parameter curve fit from the realtime fluorescence curve for the test1 primer and forward primer reaction; this is similar to the Ct value in the BioRad CFX software (though the CFX software manual does not indicate how their Ct is calculated).

*amount_gblockminusIREonlyexon* = the input gBlock amount, in picograms; in Table 1 this gBlock sequence is labeled “bBlock onlyexonNoIRE”. This gBlock is expected to be a template for the all_products reaction only.

*all_productsCp1* = first derivative of the five-parameter curve fit from the realtime fluorescence curve for the all_products primer and forward primer reaction on the gblock onlyexonNoIRE template.

*50_50gblocks* = amount of input gBlocks for the all_products reaction, since both gBlocks are amplified (200, 20….)

*test1Cp150_50* = reaction carried out with each gBlock and the test1 primers; note that the input amount is given in the amount_gBlock+ire column (100, 10…).

*allproductsCp1_50_50* = reaction carried out each gBlock and the all_products primers; note that the input amount is given in the 50_50gblocks column (200, 20…)

Supplemental Results File4 calibrated 5050_Adjustment.xlsx

The first eight columns are the same as the Supplemental Results File3 Neill2018calibrations.xlsx file. The columns with adjustments for reaction efficiency are added:

50_50_Adjustment = amount of Cp value to be changed based on the 50_50 reaction slope

Adjusted_test1 = Cp value from test1Cp150_50 column plus the 50_50_Adjustment value

Adjusted_diff = subtraction to check quality of adjustment (expected value is zero)

Supplemental Results File5 16Nov_Neill_Thesis_RNA_Adjusted.xlsx

**Temperature** = the temperature applied during the differential portion of the experiment, where “hot” refers to the high-temperature chamber and “normal” refers to the control chamber with an unchanged temperature regime

**Treatment** = plant growth regulator application code, with the untreated control named as “no hormone”. Abbreviations are paclobutrazol (PAC), propiconazole (PCZ). All plant growth regulators were applied at 50 ppm, with 10 milliliters applied to each plant pot.

**tube_Identifier_ARQ_RNA** = leaf sample unique ID, with the treatment abbreviation, underscore, replicate (as a letter of the alphabet), underscore, then a unique sample number.

**comments** = sample volume notes from sample RNA extraction

**test1Plate_Well** = realtime PCR plate well code, corresponds to the well number in the Supplemental Methods File2 7 Mar 2018 realtime plate map.doc file.

**test1_Cp1** = first derivative of the five-parameter curve fit from the realtime fluorescence curve for the test1 primer and forward primer reaction

**all_productsPrimersPlate_Well** = realtime PCR plate well code, corresponds to the well number in the Supplemental Methods File2 7 Mar 2018 realtime plate map.doc file.

**all_productsPrimers_Cp1** = first derivative of the five-parameter curve fit from the realtime fluorescence curve for the all_products primer and forward primer reaction

**product_difference** = absolute value of difference between the test1_Cp1 and the all_productsPrimers_Cp1 values

**adjustment_value** = amount needed to adjust test1 reactions to equivalent efficiency of all_products reactions; no adjustment was made if the raw value Cp was 40 (ie undetectable or zero amount of template)

**test1_adjusted** = sum of adjustment_value and test1_Cp1 values

**dif_adjusted** = difference between test1_adjusted and all_products Cp values

**femtograms allproduct** = calibration input amount was converted from picograms to femtograms and the Cp value converted to the femtogram scale

**femtograms adjtest1** = calibration input amount was converted from picograms to femtograms and the Cp value converted to the femtogram scale

**difference in femtograms** = difference between the two reaction products on the femtogram scale

**fg_diff_interpolated** = femtogram amounts were median interpolated within replicate groups to adjust for the missing values due to Cp being equal to 40. Only one interpolation was made even if multiple values were missing.

Supplemental Results File6 Full model fit details.docx

Full output of JMP analysis, with diagnostics and model fit details for all measured gene expression and plant traits.

Supplemental Results File7 summary statistics all_products. xlsx

Values of the quantile coefficient of dispersion ((75^th^ quantile minus 25^th^ quantile divided by2) divided by median Quantile) and values of the median absolute dispersion (MAD) for each of three replicate PCR reactions using the all_products primer set and both gblock templates.

Supplemental Results File 8 summary statistics test1.xlsx

Values of the quantile coefficient of dispersion ((75^th^ quantile minus 25^th^ quantile divided by2) divided by median Quantile) and values of the median absolute dispersion (MAD) for each of three replicate PCR reactions using the test1 primer set and the gblock plus18IRE template.

Supplemental Results File 9 Figure S1

Fig. S1a shows the all_products primer set and quantile coefficient of determination values across the range of concentrations of the gblock plus18IRE template. Fig S1b shows the test1 primer set and median absolute deviation (MAD) across the range of input gBlock concentrations. Fig. S1c shows the quantile coefficient of determination values across the range of concentrations for the test1 product.

Supplemental Results File 10 Supplemental Table A

This table includes the Cp values used to calculate the slopes provides in Table 2, and the Cp values used to calculate the false positive rate (specificity) for the test1 primerset.
